# Shock index-modified qSOFA improves mortality risk stratification in patients with sepsis beyond conventional qSOFA

**DOI:** 10.3389/fmed.2026.1908838

**Published:** 2026-08-31

**Authors:** Yue Zhou, Yue-feng He, Jian Rao, Jin-long Wang

**Affiliations:** 1 Graduate Training Base of Jinzhou Medical University (Chongqing University Fuling Hospital), Chongqing, China; 2 Department of Critical Care Medicine, Dujiangyan People’s Hospital, Chengdu, Sichuan, China; 3 Department of Endocrinology and Metabolism, Dujiangyan People’s Hospital, Chengdu, Sichuan, China; 4 Department of Emergency, Chongqing University Fuling Hospital, Chongqing University, Chongqing, China

**Keywords:** mortality prediction, qSOFA, risk stratification, sepsis, shock index

## Abstract

**Background:**

The quick Sequential Organ Failure Assessment (qSOFA) score does not incorporate hemodynamic perfusion parameters, which may limit its ability to identify septic patients with early circulatory compromise before the development of overt hypotension. This study aimed to evaluate a modified score incorporating the shock index (SI), termed SI-qSOFA, and to determine whether it could improve identification of high-risk patients classified as low-risk by conventional qSOFA.

**Methods:**

A retrospective cohort study was conducted, including 2,853 adult patients with sepsis. The primary outcome was in-hospital mortality, while ICU admission was treated as a secondary, exploratory outcome. In addition to evaluating overall predictive performance, a prespecified subgroup analysis was performed in patients with qSOFA < 2 to determine whether SI-qSOFA could identify high-risk patients who would otherwise have been considered low-risk. Discriminative ability was assessed using receiver operating characteristic (ROC) curves. Multivariable logistic regression models were constructed to evaluate independent associations. Calibration was assessed using the bootstrap resampling. Clinical utility was evaluated using decision curve analysis (DCA). Net reclassification improvement (NRI) and integrated discrimination improvement (IDI) were calculated to assess incremental predictive value.

**Results:**

Among the 2,853 patients, 2,456 (86.1%) had qSOFA < 2. Within this conventionally low-risk subgroup, SI-qSOFA demonstrated good discriminative performance for in-hospital mortality (AUC = 0.79, 95% CI 0.75–0.83) and ICU admission (AUC = 0.83, 95% CI 0.80–0.86), with negative predictive values of 0.98 for both outcomes. In the overall cohort, SI-qSOFA showed slightly higher discrimination than qSOFA for in-hospital mortality (AUC = 0.81 vs. 0.79, DeLong *P* = 1.78 × 10^−6^) and ICU admission (AUC = 0.85 vs. 0.82, DeLong *P* = 2.48 × 10^−11^). In multivariable analyses, SI-qSOFA remained independently associated with both outcomes. Reclassification analyses demonstrated modest improvement in partially adjusted models; however, incremental value was attenuated in the fully adjusted model for mortality. Calibration was comparable between the two scores. Decision curve analysis indicated a slightly greater net benefit of SI-qSOFA across certain threshold probabilities, particularly for ICU admission.

**Conclusion:**

SI-qSOFA may improve identification of septic patients with occult circulatory dysfunction who are classified as low-risk by conventional qSOFA. Although the overall improvement in discrimination was modest, SI-qSOFA consistently identified additional high-risk patients within the qSOFA-negative population while maintaining simplicity for bedside application. These findings suggest that SI-qSOFA may serve as a practical adjunctive bedside tool for early sepsis risk stratification and outcome prediction before the development of overt hypotension.

## Introduction

Sepsis is a life-threatening syndrome characterized by a dysregulated host response to infection, leading to acute organ dysfunction and high global mortality (1). Despite advances in critical care, sepsis continues to impose a considerable global health burden. The Global Burden of Disease study reported that sepsis-related deaths account for 31.5% of all global deaths, with increasing incidence and mortality among adults, particularly in aging populations (2). Therefore, early identification of patients at high risk of clinical deterioration is essential for guiding timely intervention and improving outcomes.

The Sepsis-3 consensus introduced the quick Sequential Organ Failure Assessment (qSOFA) score, comprising respiratory rate, systolic blood pressure, and mental status, as a rapid bedside tool for risk stratification in patients with suspected infection (3). Although qSOFA is simple and widely used in emergency departments and general wards (4), its sensitivity for early sepsis recognition remains limited, particularly in patients with compensated circulatory dysfunction in whom blood pressure is preserved despite evolving hemodynamic instability. Hwang et al. reported a sensitivity of only 39% for mortality prediction among patients with sepsis (5), and a meta-analysis further demonstrated that qSOFA has substantially lower sensitivity than other commonly used scoring systems (6). Consequently, a considerable proportion of high-risk patients may remain qSOFA-negative, potentially delaying appropriate escalation of monitoring and treatment. Therefore, an improved bedside tool should enhance early risk identification while preserving the simplicity and accessibility of qSOFA.

The shock index (SI), defined as the ratio of heart rate to systolic blood pressure, is a simple hemodynamic parameter capable of detecting early circulatory abnormalities beyond conventional vital signs (7, 8). Incorporating complementary physiological indicators may enhance prognostic assessment compared with reliance on individual scoring components alone (9). Given that sepsis is characterized by dynamic alterations in cardiovascular function, integrating SI into the qSOFA framework may capture early hemodynamic disturbances that are not reflected by systolic blood pressure alone.

Although previous studies have investigated the prognostic value of SI in sepsis, most have focused on single-indicator analyses, and its integration into existing scoring systems such as qSOFA remains underexplored. Moreover, current clinical scoring tools may still be inadequate in identifying high-risk patients requiring early antibiotic therapy or escalation of care (10). Consequently, evidence on incorporating SI into qSOFA remains limited, particularly regarding comprehensive evaluation of discrimination, calibration, and clinical utility.

Accordingly, this study developed a modified scoring system (SI-qSOFA) by incorporating the shock index into the qSOFA framework. In accordance with the most recent Surviving Sepsis Campaign guidelines (SSC 2026), which emphasize early recognition, risk-stratified management, and identification of circulatory impairment rather than reliance solely on hypotension (11), we aimed to evaluate its performance for risk stratification in adult patients with sepsis, with a primary focus on in-hospital mortality and an exploratory assessment of ICU admission. The performance of SI-qSOFA was compared with that of conventional qSOFA across multiple dimensions, including discrimination, calibration, reclassification, and clinical utility. Given the ongoing need for simple bedside tools to support early sepsis risk assessment, identifying patients with occult circulatory dysfunction may provide additional information for clinical monitoring and decision-making.

## Materials and methods

### Study design and population

This was a single-center retrospective cohort study including adult patients (≥18 years) who were hospitalized at Dujiangyan People’s Hospital between August 24, 2024, and December 31, 2025, with sepsis diagnosed according to Sepsis-3 criteria, defined as suspected or confirmed infection accompanied by an increase in SOFA score ≥2 from baseline (3). The study was conducted in accordance with the Declaration of Helsinki and was approved by the institutional ethics committee of Dujiangyan People’s Hospital (Approval Number: DuRen Medical Review 2025-S-29). The requirement for informed consent was waived due to the retrospective design and the use of anonymized data.

Inclusion criteria were: (1) confirmed diagnosis of sepsis; (2) availability of complete vital sign measurements within 24 h of admission; and (3) definite clinical outcomes or completion of 28-day follow-up. Exclusion criteria included: (1) age <18 years; (2) pregnancy; (3) discharge or transfer within 24 h of admission; and (4) missing key variables exceeding 30%. Patients who were transferred from other hospitals after initial evaluation were also excluded to reduce potential bias arising from pre-admission therapeutic interventions that could influence physiological measurements.

All variables were obtained from the first recorded values within 24 h of admission, prioritizing emergency department or initial admission assessments. Exposure variables included qSOFA and SI-qSOFA scores. These values represented the earliest available measurements obtained during emergency department triage or initial ward assessment, and were recorded before fluid resuscitation or vasopressor therapy in the majority of cases. However, in a minority of patients, pre-hospital or early in-hospital interventions may have occurred before the first recorded measurement.

### Data collection and variable definitions

Baseline characteristics, vital signs, laboratory parameters, and clinical outcomes were extracted from the electronic medical record system using a standardized data collection protocol.

Demographic data included patient identifiers, admission date, sex, age, and anthropometric measurements (height and weight), from which body mass index (BMI, kg/m^2^) was calculated. Comorbidities (including atrial fibrillation) and key interventions (antibiotics, vasopressors, intubation/invasive ventilation) were collected as potential confounding variables.

Circulatory parameters (systolic blood pressure, diastolic blood pressure, heart rate) and respiratory and neurological indicators (respiratory rate, temperature, Glasgow Coma Scale [GCS]) were recorded. The shock index (SI) was calculated as heart rate divided by systolic blood pressure (SI = HR/SBP) (12).

The conventional qSOFA score was calculated based on respiratory rate ≥22/min, systolic blood pressure ≤100 mmHg, and GCS < 15. Each component was assigned 1 point, resulting in a total score ranging from 0 to 3, with a score ≥2 considered positive (3, 4). In designing the modified score, we replaced the SBP component with SI rather than adding SI as a fourth item. This approach preserves the original 0–3 scoring range of qSOFA and avoids additional scoring complexity, thereby maintaining the bedside simplicity that underpins qSOFA’s widespread clinical acceptance. The SI-qSOFA score replaced the systolic blood pressure criterion with SI > 1 while retaining the remaining qSOFA components; a total score ≥2 was defined as positive.

Laboratory parameters included complete blood count (WBC, hemoglobin, platelet count, lymphocyte count), liver and renal function (creatinine, blood urea nitrogen, total bilirubin, albumin), coagulation (INR), and inflammatory markers (CRP, procalcitonin).

The primary outcome was in-hospital all-cause mortality, which was assessed up to 28 days from admission or hospital discharge, whichever occurred first. ICU admission was treated as a secondary, exploratory outcome, as it may be influenced by clinical decision-making processes and institutional practices in addition to disease severity.

### Statistical analysis

All analyses were performed using R software (version 4.3.1).

To address missing data, we first assessed the underlying missingness mechanism. Little’s MCAR test was performed on all variables included in the multivariable models, which rejected the null hypothesis of complete random missingness (χ^2^ = 1225.61, df = 98, *P* < 0.001), indicating that the data were not missing completely at random (MCAR). Given that the highest proportion of missing data was only 17.2% and the missingness was likely dependent on observed covariates, the missing at random (MAR) assumption was considered reasonable. Under this assumption, multiple imputation by chained equations (MICE) was used to generate five imputed datasets with 20 iterations each. Analyses were conducted independently in each dataset and pooled using Rubin’s rules.

Normality of continuous variables was assessed using the Shapiro–Wilk test; because all continuous variables deviated from a normal distribution, they were presented as median (interquartile range, IQR), and categorical variables as frequencies and percentages. Group comparisons were performed using the Kruskal–Wallis test for continuous variables and chi-square or Fisher’s exact test for categorical variables. All tests were two-sided, with *P* < 0.05 considered statistically significant.

Discriminative performance was evaluated using ROC curves, with AUC values and 95% CIs calculated and comparisons between models performed using the DeLong test. Optimal cut-off values were determined using the Youden index, and sensitivity, specificity, PPV, and NPV were calculated. To further assess incremental predictive value, net reclassification improvement (NRI) and integrated discrimination improvement (IDI) were calculated comparing SI-qSOFA with qSOFA across different adjustment models.

A two-stage analytical strategy was applied. First, covariates were selected using bidirectional stepwise logistic regression (entry/removal *P* = 0.05). As a sensitivity analysis to assess the robustness of the stepwise-selected covariates, LASSO (least absolute shrinkage and selection operator) regression with 10-fold cross-validation was applied (alpha = 1), in which SI-qSOFA was forced into the model without penalization (penalty.factor = 0) while all candidate covariates were subject to L1 shrinkage; binomial deviance was used as the tuning measure. Second, three multivariable models were constructed to assess robustness. Variance inflation factors (VIF < 5) were used to assess multicollinearity.

Calibration was further quantified using the calibration slope and calibration intercept, derived from regressing the outcome on the predicted linear predictor, with bootstrap optimism correction (1,000 resamples). Clinical utility was assessed using DCA by comparing net benefit across threshold probabilities.

To evaluate whether SI-qSOFA could identify high-risk patients classified as low-risk by conventional qSOFA, we performed a prespecified subgroup analysis restricted to patients with qSOFA < 2. Discriminative performance, optimal cut-off values, sensitivity, specificity, PPV, and NPV were assessed within this subgroup. Sensitivity analyses were conducted after excluding patients receiving vasopressors or those with atrial fibrillation (*n* = 2,394). These two factors were excluded because vasopressor use and arrhythmia directly affect systolic blood pressure and heart rate and, consequently, the shock index; excluding these factors aimed to reduce potential confounding from treatment-related hemodynamic alterations and better isolate the association between SI-qSOFA and outcomes.

## Results

### Baseline characteristics

The proportion of missing data for each study variable ranged from 0% to 17.2%, with the highest rate observed for C-reactive protein (Supplementary Table 1). Little’s test supported the MAR assumption (*P* < 0.001), thereby supporting the application of multiple imputation. Among 2,853 patients, 196 (6.9%) died during hospitalization. Compared with survivors, non-survivors were significantly older and had higher illness severity at presentation, as indicated by higher qSOFA and SI-qSOFA scores and lower Glasgow Coma Scale scores (all *P* < 0.01). In addition, non-survivors exhibited greater physiologic instability and evidence of organ dysfunction. Overall, both qSOFA and SI-qSOFA scores showed a consistent and significant gradient across in-hospital mortality status, with higher values observed in non-survivors (Table 1, Panel A). Findings were consistent in the ICU admission analysis, where patients requiring ICU care also demonstrated significantly higher qSOFA and SI-qSOFA scores, as well as greater physiological derangement, compared with non-ICU patients (Table 1, Panel B).

**TABLE 1:** Baseline characteristics stratified by in-hospital mortality and ICU admission.

Panel A. Stratified by in-hospital mortality				
Variable	Overall (*n* = 2,853)	Survivors (*n* = 2,657)	Non-survivors (*n* = 196)	*P*-value
Sex, *n* (%)				<0.01
Male	1597 (56.0)	1462 (55.0)	135 (68.9)	
Female	1256 (44.0)	1195 (45.0)	61 (31.1)	
Age, years	58.0 (37.0–75.0)	56.0 (36.0–73.0)	79.5 (71.8–87.0)	<0.01
BMI, kg/m^2^	22.1 (19.1–25.1)	22.3 (19.5–25.2)	19.2 (15.2–22.0)	<0.01
qSOFA score	0 (0–1)	0 (0–1)	1 (1–2)	<0.01
SI-qSOFA score	0 (0–1)	0 (0–1)	1 (1–2)	<0.01
Shock index	0.75 (0.63–0.91)	0.74 (0.63–0.89)	0.81 (0.67–1.03)	<0.01
GCS score	15 (15–15)	15 (15–15)	13 (8.75–14)	<0.01
SBP, mmHg	118 (103–133)	117 (103–132)	121 (105–139)	0.02
DBP, mmHg	72 (63–81)	72 (63–81)	69 (59.8–78)	<0.01
Heart rate, bpm	88 (78–102)	87 (78–100)	100 (85.8–118)	<0.01
Respiratory rate, breaths/min	20 (20–21)	20 (20–21)	21 (20–24)	<0.01
Temperature, °C	36.7 (36.5–37.0)	36.7 (36.5–37.0)	36.7 (36.5–37.1)	0.14
WBC, ×10^9^/L	9.28 (6.43–12.90)	9.35 (6.43–12.90)	8.71 (6.34–13.70)	0.78
Hemoglobin, g/L	130 (113–145)	131 (115–146)	100 (81.8–125)	<0.01
Platelet, ×10^9^/L	176 (131–234)	176 (133–235)	154 (103–223)	<0.01
Creatinine, μmol/L	67.1 (52.2–87.7)	65.8 (51.9–84.4)	108.0 (73.1–189.0)	<0.01
BUN, mmol/L	5.61 (4.24–7.97)	5.45 (4.18–7.52)	10.9 (6.63–18.20)	<0.01
Total bilirubin, μmol/L	14.0 (9.62–20.40)	14.1 (9.77–20.50)	12.6 (8.00–18.30)	0.01
INR	1.17 (1.09–1.31)	1.16 (1.08–1.29)	1.29 (1.16–1.55)	<0.01
CRP, mg/L	23.6 (10.0–89.9)	22.2 (10.0–86.1)	53.3 (15.6–141.0)	<0.01
PCT, ng/mL	0.20 (0.07–0.78)	0.19 (0.07–0.71)	0.54 (0.24–1.83)	<0.01
Lymphocyte, ×10^9^/L	0.95 (0.56–1.48)	0.98 (0.58–1.51)	0.66 (0.39–1.11)	<0.01
Albumin, g/L	40.5 (34.8–44.5)	40.9 (35.8–44.8)	32.1 (27.8–36.5)	<0.01
Antibiotic use, *n* (%)				0.02
Yes	2,706 (94.8)	2,513 (94.6)	193 (98.5)	
No	147 (5.2)	144 (5.4)	3 (1.5)	
Vasopressor use, *n* (%)				<0.01
Yes	362 (12.7)	235 (8.8)	127 (64.8)	
No	2,491 (87.3)	2,422 (91.2)	69 (35.2)	
Atrial fibrillation, *n* (%)				<0.01
Yes	148 (5.2)	110 (4.1)	38 (19.4)	
No	2,705 (94.8)	2,547 (95.9)	158 (80.6)	
Intubation, *n* (%)				<0.01
Yes	276 (9.7)	194 (7.3)	82 (41.8)	
No	2,577 (90.3)	2,463 (92.7)	114 (58.2)	
**Panel B. Stratified by ICU admission**				
**Variable**	**Overall (*n* = 2,853)**	**Non-ICU (*n* = 2,461)**	**ICU (*n* = 392)**	***P*-value**
Sex, *n* (%)				<0.01
Male	1597 (56.0)	1348 (54.8)	249 (63.5)	
Female	1256 (44.0)	1113 (45.2)	143 (36.5)	
Age, years	58.0 (37.0–75.0)	55.0 (34.0–73.0)	75.0 (64.8–83.0)	<0.01
Height, cm	160 (154–169)	160 (154–169)	160 (155–170)	0.14
Weight, kg	58.0 (47.5–67.0)	59.0 (48.0–67.5)	56.5 (46.8–65.0)	0.2
BMI, kg/m^2^	22.1 (19.1–25.1)	22.2 (19.2–25.2)	21.0 (19.0–24.0)	<0.01
qSOFA score	0 (0–1)	0 (0–1)	1 (1–2)	<0.01
SI-qSOFA score	0 (0–1)	0 (0–1)	1 (1–2)	<0.01
Shock index	0.75 (0.63–0.91)	0.74 (0.63–0.89)	0.82 (0.65–1.04)	<0.01
GCS score	15 (15–15)	15 (15–15)	13 (8–14)	<0.01
SBP, mmHg	118 (103–133)	116 (102–131)	124 (107–141)	<0.01
DBP, mmHg	72 (63–81)	72 (63–81)	72 (62–81)	0.55
Heart rate, bpm	88 (78–102)	86 (77–99)	102 (86–119)	<0.01
Respiratory rate, breaths/min	20 (20–21)	20 (20–21)	21 (20–24)	<0.01
Temperature, °C	36.7 (36.5–37.0)	36.7 (36.5–36.9)	36.8 (36.6–37.2)	<0.01
WBC, ×10^9^/L	9.28 (6.43–12.90)	9.12 (6.34–12.70)	10.30 (6.96–14.80)	<0.01
Hemoglobin, g/L	130 (113–145)	131 (116–146)	118 (94–139)	<0.01
Platelet, ×10^9^/L	176 (131–234)	177 (134–237)	158 (108–218)	<0.01
Creatinine, μmol/L	67.1 (52.2–87.7)	64.7 (51.2–81.7)	92.8 (68.6–167.0)	<0.01
BUN, mmol/L	5.61 (4.24–7.97)	5.36 (4.14–7.34)	8.39 (5.64–14.80)	<0.01
Total bilirubin, μmol/L	14.0 (9.62–20.40)	14.2 (9.81–20.80)	12.7 (8.72–18.30)	<0.01
INR	1.17 (1.09–1.31)	1.16 (1.08–1.29)	1.22 (1.11–1.40)	<0.01
CRP, mg/L	23.6 (10.0–89.9)	21.3 (10.0–82.5)	47.4 (10.0–148.0)	<0.01
PCT, ng/mL	0.20 (0.07–0.78)	0.18 (0.07–0.66)	0.51 (0.12–3.20)	<0.01
Lymphocyte, ×10^9^/L	0.95 (0.56–1.48)	0.99 (0.59–1.52)	0.76 (0.41–1.20)	<0.01
Albumin, g/L	40.5 (34.8–44.5)	41.2 (36.0–44.9)	35.3 (30.4–39.9)	<0.01
Antibiotic use, *n* (%)				<0.01
Yes	2706 (94.8)	2317 (94.1)	389 (99.2)	
No	147 (5.2)	144 (5.9)	3 (0.8)	
Vasopressor use, *n* (%)				<0.01
Yes	362 (12.7)	112 (4.6)	250 (63.8)	
No	2,491 (87.3)	2,349 (95.4)	142 (36.2)	
Atrial fibrillation, *n* (%)				<0.01
Yes	148 (5.2)	105 (4.3)	43 (11.0)	
No	2,705 (94.8)	2,356 (95.7)	349 (89.0)	
Intubation, *n* (%)				<0.01
Yes	276 (9.7)	14 (0.6)	262 (66.8)	
No	2,577 (90.3)	2,447 (99.4)	130 (33.2)	
ICU days	0 (0–0)	0 (0–0)	4 (2–7)	<0.01
In-hospital mortality, *n* (%)				<0.01
Yes	196 (6.9)	98 (4.0)	98 (25.0)	
No	2,657 (93.1)	2,363 (96.0)	294 (75.0)	
Hospital stay, days	7.0 (5.0–11.0)	7.0 (5.0–10.0)	10.0 (4.0–18.0)	<0.01

Data are presented as median (interquartile range) for continuous variables and number (percentage) for categorical variables. *P*-values were calculated using the Kruskal-Wallis H test for continuous variables and the Chi-square test or Fisher’s exact test for categorical variables, as appropriate. In-hospital mortality was defined as all-cause death occurring during hospitalization, assessed up to 28 days from admission or hospital discharge, whichever occurred first. BMI, body mass index; qSOFA, quick Sequential Organ Failure Assessment; SI, shock index; GCS, Glasgow Coma Scale; SBP, systolic blood pressure; DBP, diastolic blood pressure; WBC, white blood cell count; BUN, blood urea nitrogen; INR, international normalized ratio; CRP, C-reactive protein; PCT, procalcitonin; ICU, intensive care unit.

### Performance of SI-qSOFA in patients with qSOFA < 2

Among the 2,853 included patients, 2,456 (86.1%) had qSOFA < 2 and would therefore be classified as low-risk by conventional qSOFA criteria. Within this subgroup, the in-hospital mortality rate remained 4.44%, while the ICU admission rate was 9.12%. ROC curve analysis demonstrated that SI-qSOFA retained good discriminative performance despite conventional qSOFA negativity. For in-hospital mortality, the AUC was 0.79 (95% CI: 0.75–0.83); for ICU admission, the AUC was 0.83 (95% CI: 0.80–0.86), with bootstrap-corrected *P*-values of 8.00 × 10^−9^ and 9.82 × 10^−9^, respectively. Using the Youden index, the optimal cut-off value for SI-qSOFA was 0.5 (corresponding to a score ≥ 1) for both outcomes. The optimal threshold corresponded to SI-qSOFA ≥ 1, suggesting its potential value for identifying higher-risk patients within the qSOFA-negative subgroup. At this threshold, SI-qSOFA achieved high sensitivity and negative predictive value for both in-hospital mortality (sensitivity 0.91; NPV 0.98) and ICU admission (sensitivity 0.88; NPV 0.98) (Table 2, Panel B). These findings indicate that SI-qSOFA may identify additional patients at elevated risk within the qSOFA-negative subgroup.

**TABLE 2:** Discriminative performance of SI-qSOFA across the overall cohort, qSOFA-negative subgroup, and sensitivity cohort.

Panel A. Overall cohort (*N* = 2,853)					
Outcome	Metric	qSOFA	SI-qSOFA	DeLong *P*	Bootstrap *P*
In-hospital mortality	AUC (95% CI)	0.79 (0.76–0.82)	0.81 (0.79–0.84)	1.78 × 10^−6^	3.28 × 10^−6^
	Cut-off value	0.5	0.5	–	
	Youden index	0.5	0.56	–	
	Sensitivity	0.9	0.9	–	
	Specificity	0.6	0.66	–	
	PPV	0.14	0.16	–	
	NPV	0.98	0.98	–	
ICU admission	AUC (95% CI)	0.82 (0.80–0.84)	0.85 (0.83–0.87)	2.48 × 10^−11^	3.16 × 10^−11^
	Cut-off value	0.5	0.5	–	
	Youden index	0.57	0.65	–	
	Sensitivity	0.92	0.93	–	
	Specificity	0.65	0.72	–	
	PPV	0.29	0.34	–	
	NPV	0.98	0.98	–	
**Panel B. Patients with qSOFA < 2 (*N* = 2,456)**					
In-hospital mortality	AUC (95% CI)	–	0.79 (0.75–0.83)	–	8.00 × 10^−9^*
	Cut-off value	–	0.5	–	
	Youden index	–	0.64	–	
	Sensitivity	–	0.91	–	
	Specificity	–	0.73	–	
	PPV	–	0.22	–	
	NPV	–	0.98	–	
ICU admission	AUC (95% CI)	–	0.83 (0.80–0.86)	–	9.82 × 10^−9^*
	Cut-off value	–	0.5	–	
	Youden index	–	0.67	–	
	Sensitivity	–	0.88	–	
	Specificity	–	0.79	–	
	PPV	–	0.29	–	
	NPV	–	0.98	–	
**Panel C. Sensitivity analysis (Excluding vasopressor use or arrhythmia, *N* = 2,394)**					
In-hospital mortality	AUC (95% CI)	0.78 (0.72–0.84)	0.81 (0.76–0.87)	8 × 10^−4^	8 × 10^−4^
	Cut-off value	0.5	0.5	–	
	Youden index	0.5	0.59	–	
	Sensitivity	0.84	0.86	–	
	Specificity	0.66	0.73	–	
	PPV	0.06	0.07	–	
	NPV	0.99	0.99	–	
ICU admission	AUC (95% CI)	0.82 (0.79–0.85)	0.85 (0.82–0.88)	4.68 × 10^−14^	1.77 × 10^−13^
	Cut-off value	0.5	0.5	–	
	Youden index	0.6	0.66	–	
	Sensitivity	0.92	0.92	–	
	Specificity	0.68	0.74	–	
	PPV	0.13	0.17	–	
	NPV	0.99	0.99	–	

*DeLong *P*-values were calculated for comparisons between qSOFA and SI-qSOFA AUCs; Bootstrap *P*-values (1,000 iterations) were obtained from internal validation. In Panel B, only SI-qSOFA was assessed, and the reported Bootstrap *P*-value reflects the stability of the AUC estimate. AUC, area under the receiver operating characteristic curve; CI, confidence interval; PPV, positive predictive value; NPV, negative predictive value. Cut-off values were determined using the Youden index. –: Not applicable.

### Discriminative performance and reclassification

**FIGURE 1:**
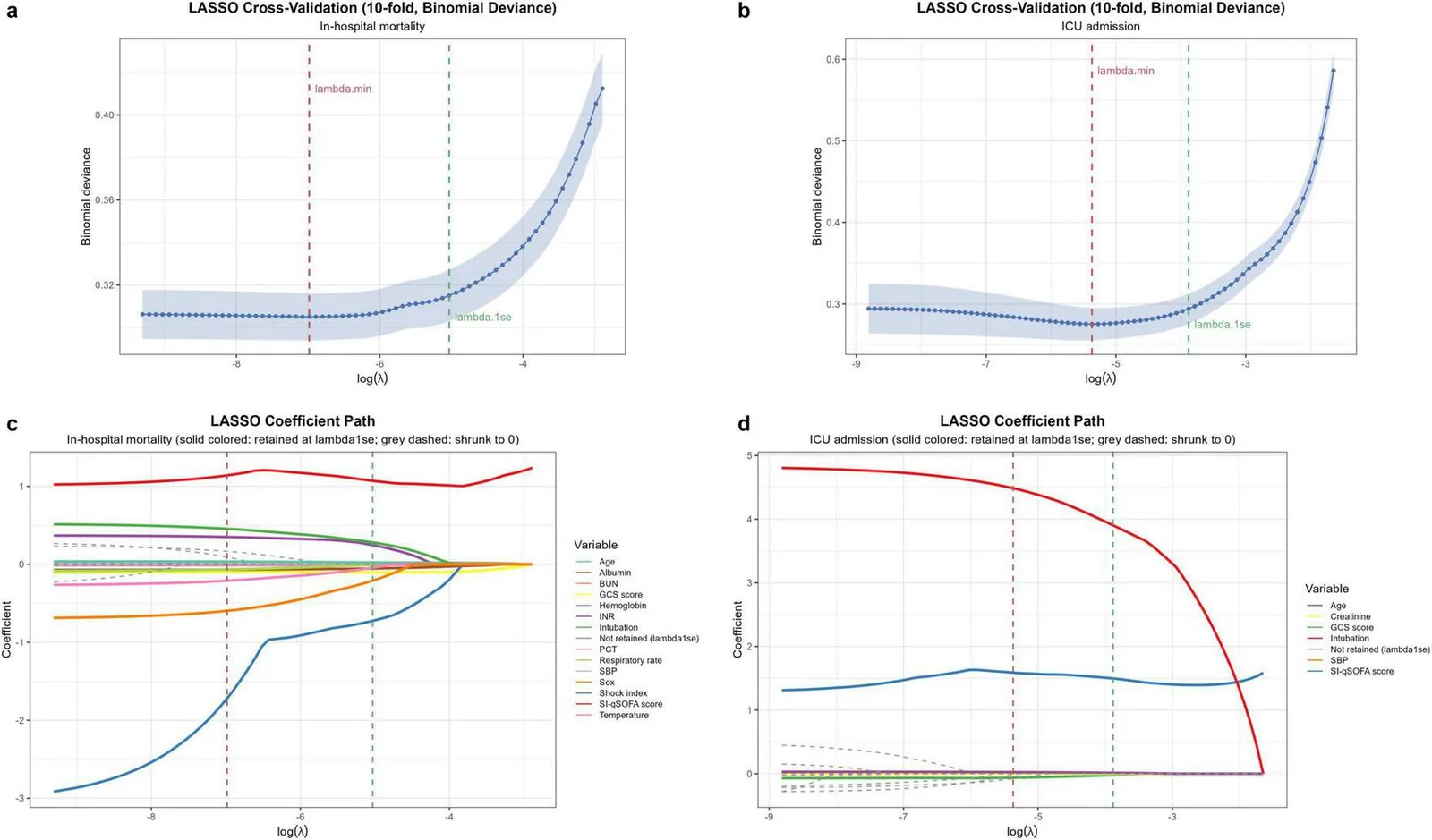
Comparison of the discriminative performance of qSOFA and SI-qSOFA scores. Receiver operating characteristic (ROC) curves for predicting **(a)** ICU admission and **(b)** in-hospital mortality. The area under the curve (AUC) values with 95% confidence intervals (CIs) are displayed in the legend. SI-qSOFA showed significantly higher AUC values compared to qSOFA in both outcomes (*P* < 0.01).

Receiver operating characteristic curve analysis showed that SI-qSOFA had slightly improved discriminative performance compared with qSOFA for both in-hospital mortality and ICU admission. For in-hospital mortality, SI-qSOFA yielded an AUC of 0.81 (95% CI 0.79–0.84) versus 0.79 (0.76–0.82) for qSOFA (DeLong *P* = 1.78 × 10^−6^; bootstrap *P* = 3.28 × 10^−6^). For ICU admission, the corresponding AUCs were 0.85 (0.83–0.87) and 0.82 (0.80–0.84), respectively (DeLong *P* = 2.48 × 10^−11^; bootstrap *P* = 3.16 × 10^−11^) (Figure 1). Similar findings were observed in the sensitivity cohort and among patients with qSOFA < 2 (Table 2).

Reclassification analyses demonstrated a modest incremental improvement in risk classification with SI-qSOFA compared with qSOFA. For in-hospital mortality, a small but significant improvement was observed only in Model 2 (NRI = 0.021, *P* < 0.01), while no consistent benefit was observed in Model 3. For ICU admission, SI-qSOFA showed significant gains in both NRI and IDI in Model 2 (NRI = 0.034, IDI = 0.026; both *P* < 0.01), with attenuated improvement in Model 3 (Supplementary Table 2).

Based on the optimal cut-off values determined by the Youden index, SI-qSOFA demonstrated better discriminative performance than qSOFA for both in-hospital mortality (Youden index: 0.56 vs. 0.50) and ICU admission (0.65 vs. 0.57). For in-hospital mortality, sensitivities were comparable (both 0.90), whereas SI-qSOFA showed higher specificity (0.66 vs. 0.60) and positive predictive value (0.16 vs. 0.14). For ICU admission (exploratory outcome), SI-qSOFA had slightly higher sensitivity (0.93 vs. 0.92), higher specificity (0.72 vs. 0.65), and higher positive predictive value (0.34 vs. 0.29). Both scores had similarly high negative predictive values (0.98 for both outcomes) (Table 2, Panel A). These findings indicate that SI-qSOFA provided modest improvements in classification performance compared with qSOFA, although the absolute gains were limited.

### Multivariable association

To determine whether these associations were independent of potential confounders, three progressively adjusted multivariable models were constructed: Model 1 included only the score; Model 2 additionally adjusted for age and sex; Model 3 further included temperature, TBil, INR, CRP, and intubation status.

In the full cohort, the SI-qSOFA score was significantly associated with outcomes across all progressively adjusted models. In the fully adjusted model (Model 3), each 1-point increase in SI-qSOFA was associated with a significantly higher risk of in-hospital mortality (OR = 3.30, 95% CI: 2.75–3.98, *P* < 0.01) and of ICU admission (OR = 6.59, 95% CI: 5.47–8.02, *P* < 0.01). In comparison, the effect sizes for qSOFA were smaller in Model 3 (in-hospital mortality: OR = 2.96; ICU admission: OR = 4.44; both *P* < 0.01) (Table 3). In the LASSO sensitivity analysis, SI-qSOFA was retained at both λ.min and λ.1se for in-hospital mortality (24 and 14 covariates selected, respectively) and for ICU admission (11 and 6 covariates selected, respectively), with consistently positive coefficients (approximately 1.07 for mortality and 1.50 for ICU admission at λ.1se), supporting the robustness of the observed association. The covariate sets selected by LASSO and stepwise regression partially overlapped (concordance: 0.56 for mortality and 0.64 for ICU admission), which was consistent with the expected methodological differences between the two approaches (Supplementary Figure 1).

**TABLE 3:** Multivariable logistic regression analyses in the overall cohort and sensitivity cohort.

Cohort	Model	Outcome	qSOFA OR (95% CI)	*P*-value	SI-qSOFA OR (95% CI)	*P*-value
Overall cohort (*n* = 2,853)	Model 1	In-hospital mortality	3.56 (3.00–4.25)	<0.01	3.44 (2.94–4.04)	<0.01
		ICU admission	4.63 (4.00–5.40)	<0.01	4.88 (4.24–5.56)	<0.01
	Model 2	In-hospital mortality	3.45 (2.87–4.18)	<0.01	3.19 (2.69–3.79)	<0.01
		ICU admission	5.68 (4.77–6.81)	<0.01	5.57 (4.73–6.62)	<0.01
	Model 3	In-hospital mortality	2.96 (2.41–3.66)	<0.01	3.30 (2.75–3.98)	<0.01
		ICU admission	4.44 (3.53–5.65)	<0.01	6.59 (5.47–8.02)	<0.01
Sensitivity cohort (*n* = 2,394)	Model 1	In-hospital mortality	3.93 (2.85–5.42)	<0.01	3.91 (2.95–5.20)	<0.01
		ICU admission	4.71 (3.72–5.97)	<0.01	5.05 (4.05–6.30)	<0.01
	Model 2	In-hospital mortality	4.49 (3.17–6.35)	<0.01	3.90 (2.89–5.27)	<0.01
		ICU admission	7.27 (5.42–9.76)	<0.01	6.61 (5.07–8.61)	<0.01
	Model 3	In-hospital mortality	4.69 (3.22–6.84)	<0.01	4.25 (3.04–5.96)	<0.01
		ICU admission	7.51 (5.13–11.01)	<0.01	7.89 (5.45–11.44)	<0.01

OR, odds ratio; CI, confidence interval; ICU, intensive care unit. Model 1: unadjusted. Model 2: adjusted for age and sex. Model 3: further adjusted for temperature, total bilirubin (TBIL), international normalized ratio (INR), C-reactive protein (CRP), and intubation status. Sensitivity cohort: patients after excluding those receiving vasopressors or with arrhythmia.

### Calibration and clinical utility

**FIGURE 2:**
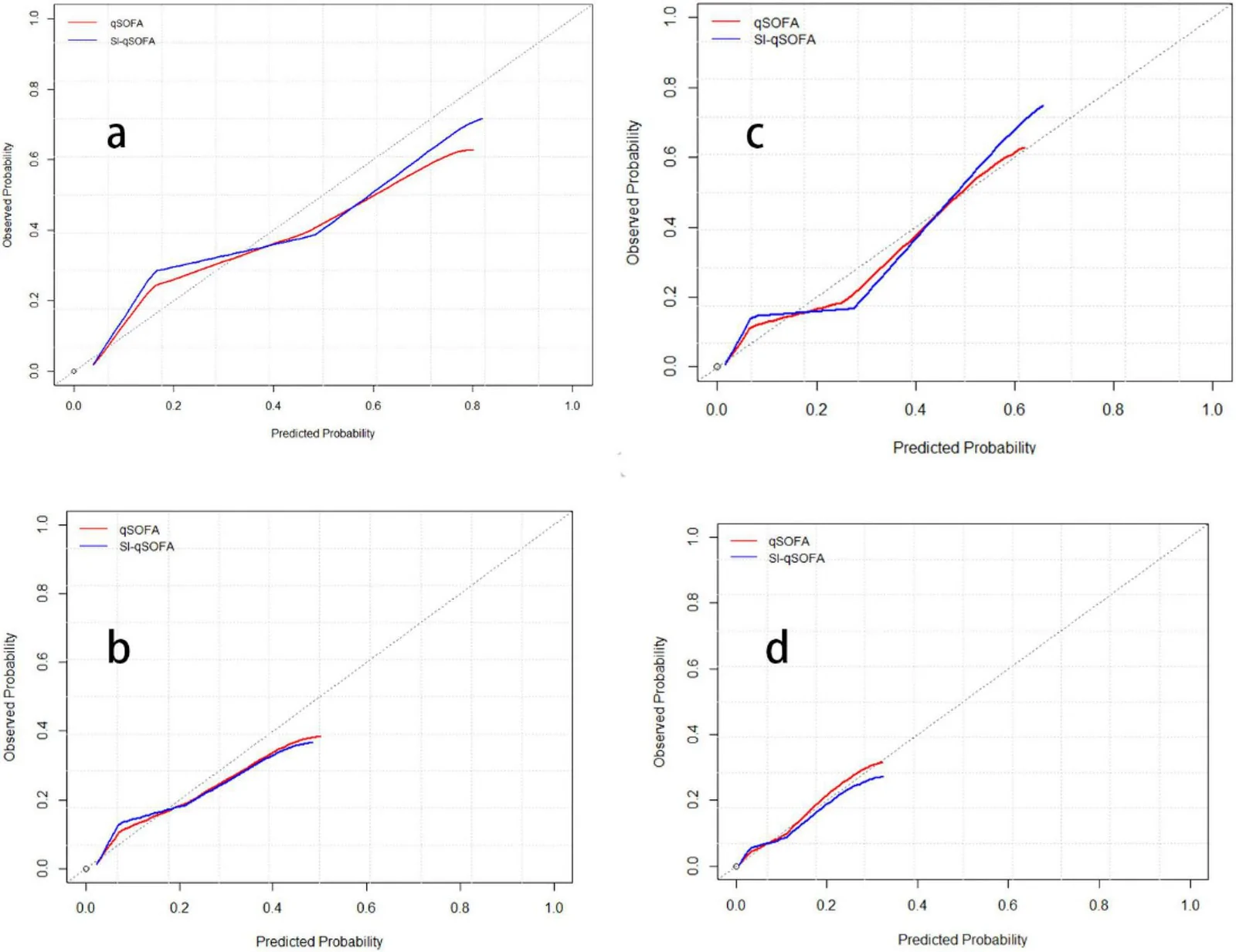
Calibration curves for the predictive models in the full cohort and subgroup. The plots compare the predicted probability (*X*-axis) against the observed probability (*Y*-axis) for panels **(a,c)** ICU admission and **(b,d)** in-hospital mortality. The 45-degree dashed line represents perfect calibration. **(a,b)** Represent the full sample analysis, while **(c,d)** represent the sensitivity analysis subgroup.

Calibration analysis showed generally good agreement between predicted and observed risks for both scoring systems. For in-hospital mortality, both qSOFA and SI-qSOFA demonstrated acceptable calibration across the observed risk range. For ICU admission (exploratory outcome), SI-qSOFA showed closer alignment with the ideal calibration line, particularly in the intermediate-risk range, whereas qSOFA tended to underestimate risk at higher probabilities (Figure 2).

For both scores, the calibration slope and intercept approximated 1.0 and 0, respectively, for in-hospital mortality (slope: qSOFA 1.000 [95% CI 0.863–1.137], SI-qSOFA 1.000 [0.872–1.128]; intercept: 0.000 [-0.154 to 0.154] and 0.000 [-0.156 to 0.156]) and for ICU admission (slope: 1.000 [0.902–1.098] and 1.000 [0.909–1.091]; intercept: -0.000 [-0.121 to 0.121] and -0.000 [-0.125 to 0.125]). Bootstrap optimism-corrected values remained close to 1.0 and 0, respectively, indicating adequate calibration.

**FIGURE 3:**
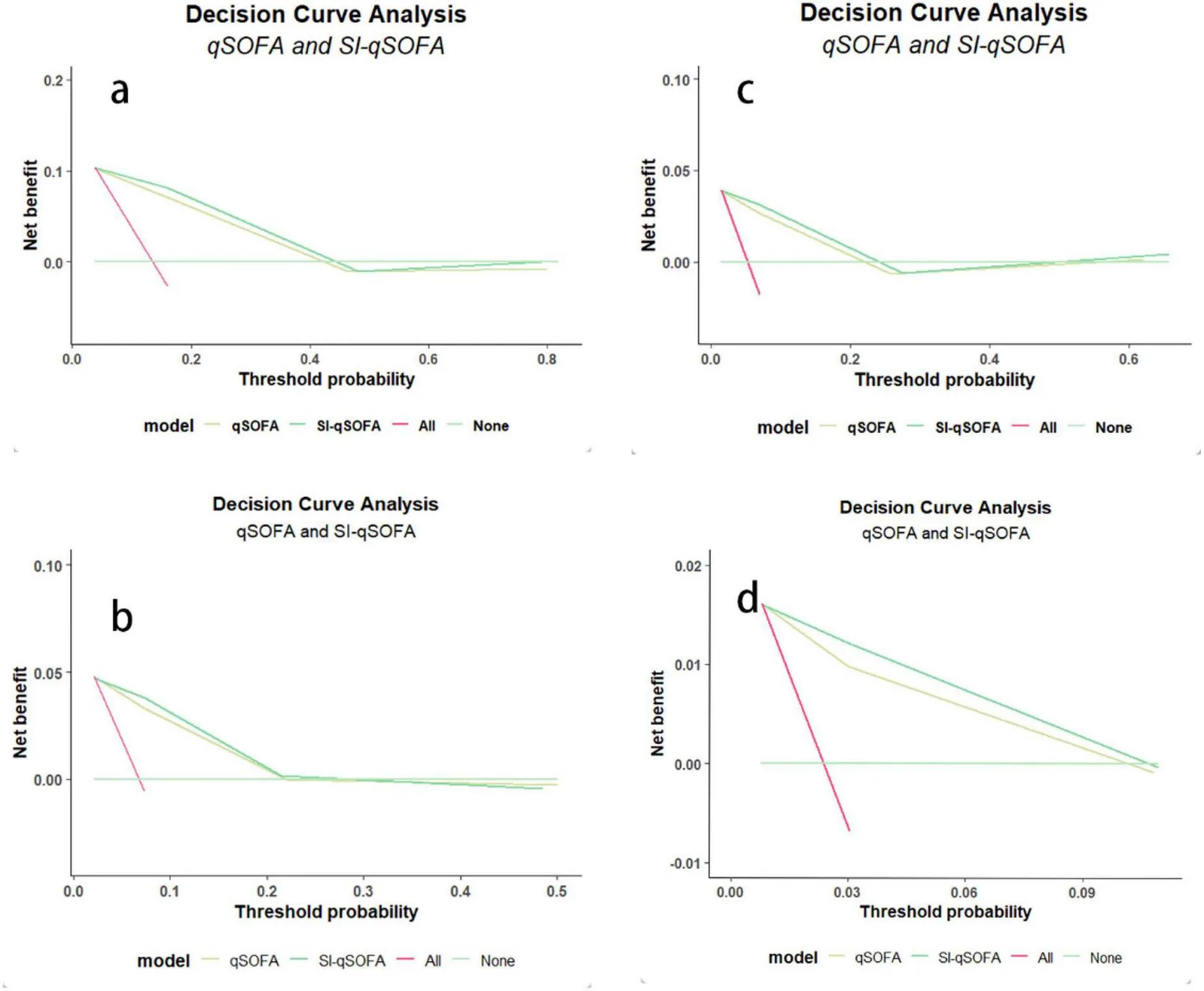
Decision curve analysis (DCA) comparing the clinical utility of qSOFA and SI-qSOFA. The net benefit of using qSOFA (red line) and SI-qSOFA (blue line) is plotted across a range of threshold probabilities. **(a,b)** Represent the analysis for the full cohort, while **(c,d)** represent the sensitivity analysis for the subgroup. **(a,c)** Show the results for ICU admission. **(b,d)** Show the results for In-hospital mortality. The gray line represents the assumption that “All” patients are treated, and the horizontal green line represents “None” (no patients are treated).

Decision curve analysis indicated that, for in-hospital mortality, both scores yielded similar and modest net benefit at low thresholds, with largely overlapping curves. For ICU admission, SI-qSOFA demonstrated a greater estimated net clinical benefit than qSOFA across threshold probabilities of approximately 0.05–0.45 (Figure 3).

### Overall predictive accuracy (Brier scores)

We further quantified overall predictive accuracy using Brier scores (Supplementary Table 3). In the full cohort, SI-qSOFA achieved slightly lower (better) Brier scores than qSOFA for both in-hospital mortality (0.0576 vs. 0.0584) and ICU admission (0.0917 vs. 0.0975). Relative to the null (intercept-only) model, SI-qSOFA showed a greater relative reduction for ICU admission (22.61% vs. 17.76%) than for mortality (9.96% vs. 8.77%). These findings indicate that the improvement in overall predictive accuracy was more pronounced for ICU admission, although the absolute gains remained modest.

### Sensitivity analyses

Sensitivity analyses excluding patients on vasopressors or with arrhythmias (*n* = 2,394) confirmed the primary findings. In fully adjusted models, SI-qSOFA remained significantly associated with in-hospital mortality (OR = 4.25, 95% CI 3.04–5.96, *P* < 0.01) and with ICU admission (OR = 7.89, 95% CI 5.45–11.44, *P* < 0.01) (Table 3). ROC analysis showed consistent AUC improvements for mortality (0.81 vs. 0.78) and for ICU admission (0.85 vs. 0.82) (Table 2, Panel C). Calibration was stable for both outcomes, and DCA confirmed similar net benefit for mortality, with a modest advantage of SI-qSOFA for ICU admission (Figures 2, 3).

To further explore the performance of SI-qSOFA across different hemodynamic severities, we performed a stratified analysis based on vasopressor use as a proxy for shock (Supplementary Table 4). Among patients not receiving vasopressors (non-shock subgroup, *n* = 2,491), SI-qSOFA demonstrated significantly improved discrimination over qSOFA for both in-hospital mortality (AUC: 0.821 vs. 0.792; DeLong *P* = 0.0011) and ICU admission (0.855 vs. 0.824; *P* < 0.001). However, in patients requiring vasopressors (shock subgroup, *n* = 362), the improvement was minimal and statistically non-significant for mortality (0.542 vs. 0.535; *P* = 0.613), with only borderline significance for ICU admission (0.604 vs. 0.574; *P* = 0.054). This suggests that the added value of SI-qSOFA may be primarily related to the identification of occult circulatory dysfunction among non-shock patients, whereas its incremental benefit may be attenuated once overt shock requiring vasopressor therapy has developed.

## Discussion

In this retrospective cohort study, the principal finding was that SI-qSOFA retained good predictive performance among patients with qSOFA < 2, who are conventionally classified as low-risk according to standard qSOFA criteria. Within this subgroup, SI-qSOFA achieved sound discrimination for both in-hospital mortality and ICU admission, with high negative predictive values. Although the overall improvement in AUC compared with conventional qSOFA was modest, these findings suggest that SI-qSOFA may help identify septic patients with early circulatory abnormalities among those who remain qSOFA-negative before overt hypotension develops.

The observed improvement in predictive performance may be related to the physiological properties of SI. Unlike relying solely on systolic blood pressure, SI integrates heart rate and systolic blood pressure, thereby indirectly reflecting hemodynamic status and potentially allowing earlier recognition of circulatory abnormalities before overt hypotension occurs. Notably, in this study, the optimal cutoff for SI-qSOFA was determined to be 0.5 (corresponding to a score ≥ 1) based on the Youden index, which is lower than the conventional qSOFA threshold (≥2). This lower threshold may allow SI-qSOFA to identify patients in the early phase of compensatory circulatory stress who might otherwise be overlooked. A threshold of ≥1 may therefore serve as a screening threshold for early bedside risk assessment, where high sensitivity and rapid identification of clinical deterioration are prioritized. In the pathophysiology of sepsis, compensatory mechanisms often temporarily maintain blood pressure despite underlying circulatory dysfunction, whereas SI demonstrates greater sensitivity in detecting patients at risk of deterioration (13, 14). Therefore, substituting systolic blood pressure with SI within the qSOFA framework may enhance early detection of circulatory stress without adding complexity, supporting rapid bedside risk stratification. Importantly, the lower SI-qSOFA threshold (≥1) is intended primarily as a screening trigger to prompt closer monitoring and further assessment rather than as a direct ICU admission criterion. The consistently high negative predictive values observed suggest that this threshold may help identify patients unlikely to experience adverse outcomes. Nevertheless, the potential resource implications of applying a more sensitive threshold warrant prospective evaluation in independent settings.

Although statistically significant differences in AUC were observed, the absolute improvement in discrimination was relatively small. This pattern is clinically plausible because qSOFA already captures severe physiological derangement, whereas SI primarily contributes by identifying earlier compensatory circulatory abnormalities before progression to overt hypotension. Therefore, the principal value of SI-qSOFA may lie less in substantially increasing overall discrimination and more in reducing false reassurance among patients classified as qSOFA-negative. This finding is consistent with previous studies on incremental predictive markers, where improvements in AUC alone may not fully capture enhancements in model performance (15). In terms of calibration, both qSOFA and SI-qSOFA demonstrate acceptable agreement between predicted and observed outcomes. SI-qSOFA showed somewhat improved calibration in the moderate-to-high risk range for ICU admission, although the clinical implications of this difference require further validation (16). However, calibration performance for mortality is comparable between the two scores, indicating that the added value of SI may vary depending on the outcome of interest.

Decision curve analysis, which evaluates clinical net benefit across threshold probabilities (17), indicates that SI-qSOFA was associated with greater estimated net benefit than qSOFA for ICU admission across a range of threshold probabilities. These findings suggest that the clinical utility of SI-qSOFA may depend on specific decision contexts and may be particularly relevant in scenarios requiring early identification of patients who may benefit from closer monitoring or escalation of care. From the perspective of sepsis outcome prediction, early recognition of occult circulatory dysfunction may facilitate timely risk stratification before overt hemodynamic deterioration develops. Of note, for in-hospital mortality, the DCA curves for qSOFA and SI-qSOFA were largely overlapping across most threshold probabilities, indicating that the net clinical benefit of SI-qSOFA for mortality prediction is limited to specific contexts. This contrasts with the more consistent benefit observed for ICU admission and further supports the interpretation that SI-qSOFA’s primary value lies in identifying early circulatory stress rather than predicting mortality itself.

In response to the limited predictive performance of qSOFA, various modification strategies have been proposed. Some studies have incorporated lactate (Lac-qSOFA) (18, 19) or inflammatory biomarkers (e.g., PCT, IL-8) (20), with reported improvements in mortality prediction. Other approaches have integrated additional physiological or clinical parameters to enhance risk stratification (21). Age-adjusted qSOFA has also been applied in specific populations, such as pediatric patients (22, 23), and further combined with other markers to improve performance (24). However, these approaches often require testing or increased complexity. In this regard, the 2026 Surviving Sepsis Campaign guidelines recommend NEWS, NEWS2, MEWS, or SIRS over qSOFA as a single hospital screening tool, reflecting the recognized limitations of qSOFA for bedside risk stratification (11). SI-qSOFA, which relies solely on routinely available vital signs, may represent an approach that retains the simplicity of qSOFA while incorporating additional hemodynamic information relevant to early risk assessment. This finding aligns with previous evidence that hemodynamic indices, including SI and its derivatives, have strong discriminative power in identifying high-risk patients (25). Mechanistically, SI may capture aspects of compensatory circulatory stress before overt hypotension, which is consistent with the evolving paradigm in sepsis management shifting from “hypotension detection” toward recognition of “hypoperfusion” (3). The stratified analysis by vasopressor use provides additional support for this interpretation: the improvement over qSOFA was predominantly observed in patients without vasopressor requirements, whereas performance was largely comparable in those with established shock. This pattern is consistent with the hypothesis that SI may be more informative for detecting early, compensated circulatory stress than late-stage hemodynamic collapse.

It should be noted that the predictive performance of qSOFA varies across studies. For example, a meta-analysis reported an AUC of approximately 0.78 for short-term mortality prediction (26). Such variability may be related to differences in patient populations, disease severity, and healthcare resources. In particular, ICU admission is influenced not only by disease severity but also by institutional practices and resource availability, which may affect its interpretation as an outcome. Therefore, evaluation of modified scoring tools should consider not only absolute discrimination but also relative improvements and clinical applicability in specific contexts.

Several limitations should be acknowledged. First, this was a single-center retrospective study, which may limit generalizability. Second, the overall in-hospital mortality rate in our cohort was relatively low, which may have reduced statistical power for detecting modest improvements in reclassification analyses. Third, ICU admission, as a secondary outcome, may be influenced by institutional practices and clinician judgment, introducing variability unrelated to disease severity. Fourth, analyses were based on variables collected within the first 24 h of admission and did not account for dynamic changes over time. Fifth, we lacked data on fluid volumes administered before physiological measurement, which may have influenced systolic blood pressure and the shock index. Sixth, detailed data on cardiovascular comorbidities and rate-controlling medications (e.g., beta-blockers) were unavailable and may have modified the shock index independently of sepsis severity. These limitations, together with the absence of external validation, underscore that SI-qSOFA should be considered hypothesis-generating and requires prospective evaluation in independent multicenter cohorts before potential clinical implementation.

Despite these limitations, SI-qSOFA represents a simple modification of the conventional qSOFA score that incorporates additional hemodynamic information through the shock index. While the overall improvement in discrimination was modest, the consistency of findings–particularly the identification of higher-risk patients among qSOFA-negative individuals–suggests that SI-qSOFA may serve as an adjunctive bedside risk stratification tool to support closer monitoring. However, given the limited incremental benefit and lack of external validation, SI-qSOFA should not replace conventional qSOFA at the current stage. Prospective multicenter studies are warranted to define its role in clinical decision-making.

## Conclusion

In this single-center retrospective cohort, SI-qSOFA showed good predictive performance in patients with qSOFA < 2, who are conventionally classified as low-risk according to standard qSOFA criteria. Although the overall improvement in discrimination compared with conventional qSOFA was modest, SI-qSOFA may help identify patients with early circulatory abnormalities associated with increased risk before overt hypotension develops. Given its reliance solely on routinely available vital signs without additional measurement burden, SI-qSOFA may serve as a practical adjunctive bedside tool for early sepsis risk stratification and outcome prediction, although its applicability across different healthcare settings requires further validation. Importantly, the identified threshold of SI-qSOFA ≥ 1 should be interpreted as a preliminary screening threshold and requires prospective external validation before consideration for routine clinical implementation.

## Data Availability

The raw data supporting the conclusions of this article will be made available by the authors, without undue reservation.
