# Supplementary material for: Shock index-modified qSOFA improves mortality risk stratification in patients with sepsis beyond conventional qSOFA
Source: Front Med (Lausanne). 2026 Aug 31;13:1908838. doi: 10.3389/fmed.2026.1908838 (PMC13572099; doi:10.3389/fmed.2026.1908838)
Supplement: Supplementary file 1 [file Table_1.docx]

Supplementary Table 1. Missing Data Patterns and Little's MCAR Test for Variables Included in the Multivariable Models

| **Variable** | **Included in multivariable models** | **Missing (n)** | **Missing rate (%)** |
| --- | --- | --- | --- |
| C-reactive protein (CRP) | Yes | 490 | 17.2 |
| INR | Yes | 290 | 10.2 |
| SI-qSOFA score | Yes | 257 | 9 |
| qSOFA score | Yes | 252 | 8.8 |
| Shock index | No | 248 | 8.7 |
| Systolic blood pressure | No | 242 | 8.5 |
| Diastolic blood pressure | No | 242 | 8.5 |
| Procalcitonin | No | 223 | 7.82 |
| Heart rate | No | 177 | 6.2 |
| Respiratory rate | No | 177 | 6.2 |
| Height | No | 175 | 6.13 |
| Weight | No | 174 | 6.1 |
| WBC count | No | 83 | 2.9 |
| Hemoglobin | No | 83 | 2.9 |
| Platelet count | No | 83 | 2.9 |
| Lymphocyte count | No | 83 | 2.9 |
| Albumin | No | 30 | 1.1 |
| Creatinine | No | 28 | 1 |
| Blood urea nitrogen | No | 28 | 1 |
| Total bilirubin | Yes | 28 | 1 |
| GCS score | No | 3 | 0.1 |
| Sex | Yes | 0 | 0 |
| Age | Yes | 0 | 0 |
| Temperature | Yes | 0 | 0 |
| Intubation status | Yes | 0 | 0 |
| Antibiotic use | No | 0 | 0 |
| Vasopressor use | No | 0 | 0 |
| Atrial fibrillation | No | 0 | 0 |
| ICU admission | No | 0 | 0 |
| In-hospital mortality | No | 0 | 0 |
| Length of hospital stay | No | 0 | 0 |

**Little's MCAR Test Results：**

| Test statistic | Variables | Sample size | χ² | df | *P* value | Conclusion |
| --- | --- | --- | --- | --- | --- | --- |
| Little's MCAR test | 9* | 2,853 | 1225.61 | 98 | < 0.001 | Reject MCAR; MAR assumption supported |

* Variables included in the test were: qSOFA score, SI-qSOFA score, sex, age, temperature, total bilirubin, INR, C-reactive protein, and intubation status (i.e., all variables that entered the multivariable Models 1–3).

**Supplementary Table 2**. Reclassification and discrimination improvement of SI-qSOFA over qSOFA for clinical outcomes (NRI and IDI analysis)

| **A. In-hospital mortality** | | | | |
| --- | --- | --- | --- | --- |
| Model | NRI (95% CI) | *P* value | IDI (95% CI) | *P* value |
| Model 1 | — | — | — | — |
| Model 2 | 0.021 (0.006–0.037) | <0.01 | -0.001 (-0.015–0.012) | >0.05 |
| Model 3 | -0.083 (-0.229–0.062) | >0.05 | 0.001 (-0.011–0.013) | >0.05 |
| **B. ICU admission** | | | | |
| Model | NRI (95% CI) | *P* value | IDI (95% CI) | *P* value |
| Model 1 | — | — | — | — |
| Model 2 | 0.034 (0.021–0.047) | <0.01 | 0.026 (0.014–0.039) | <0.01 |
| Model 3 | 0.081 (-0.066–0.228) | >0.05 | 0.014 (0.006–0.022) | <0.01 |

NRI: net reclassification improvement; IDI: integrated discrimination improvement. Model 1–3 refer to progressively adjusted regression models as defined in Table 1. NRI and IDI values are reported with 95% confidence intervals obtained using bootstrap resampling. Cutoff values (0.006, 0.017, 0.058 for in-hospital mortality; 0.018, 0.048, 0.134 for ICU admission) were predefined thresholds used for risk stratification in the reclassification analysis.

Supplementary Table 3. Brier Scores and Overall Predictive Accuracy of qSOFA and SI-qSOFA

| **Cohort** | **Outcome** | **Score** | **Events / N** | **Event rate(%)** | **Brier score** | **Null model Brier** | **Relative reduction (%)** |
| --- | --- | --- | --- | --- | --- | --- | --- |
| **Full cohort**  **(n = 2,853)** | In-hospital mortality | qSOFA | 196 / 2,853 | 6.87 | 0.0584 | 0.064 | 8.77 |
|  |  | SI-qSOFA | 196 / 2,853 | 6.87 | 0.0576 | 0.064 | 9.96 |
|  | ICU admission | qSOFA | 392 / 2,853 | 13.74 | 0.0975 | 0.1185 | 17.76 |
|  |  | SI-qSOFA | 392 / 2,853 | 13.74 | 0.0917 | 0.1185 | 22.61 |
| **Sensitivity cohort**  **(n = 2,394)*** | In-hospital mortality | qSOFA | 57 / 2,394 | 2.38 | 0.0222 | 0.0232 | 4.43 |
|  |  | SI-qSOFA | 57 / 2,394 | 2.38 | 0.0219 | 0.0232 | 5.85 |
|  | ICU admission | qSOFA | 128 / 2,394 | 5.35 | 0.0461 | 0.0506 | 8.89 |
|  |  | SI-qSOFA | 128 / 2,394 | 5.35 | 0.0433 | 0.0506 | 14.42 |
| **qSOFA < 2 subgroup**  **(n = 2,456)** | In-hospital mortality | qSOFA | 109 / 2,456 | 4.44 | 0.0403 | 0.0424 | 5.05 |
|  |  | SI-qSOFA | 109 / 2,456 | 4.44 | 0.0412 | 0.0424 | 2.86 |
|  | ICU admission | qSOFA | 224 / 2,456 | 9.12 | 0.0719 | 0.0829 | 13.21 |
|  |  | SI-qSOFA | 224 / 2,456 | 9.12 | 0.0748 | 0.0829 | 9.72 |

Abbreviations: Brier score, a proper scoring rule ranging from 0 to 1, with lower values indicating better overall predictive accuracy; Null model Brier, Brier score of the intercept-only (null) model, which predicts the same event rate for all patients; Relative reduction, calculated as (null model Brier – model Brier) / null model Brier × 100%. * Sensitivity cohort excluded patients receiving vasopressors or those with atrial fibrillation. There was overlap between the two exclusion criteria (patients with both vasopressor use and atrial fibrillation), yielding a final cohort of 2,394 patients.

Supplementary Table 4. Stratified Analysis of SI-qSOFA Performance by Vasopressor Use (Shock Proxy)

| **Stratum** | **Outcome** | **N** | **Events** | **qSOFA AUC (95% CI)** | **SI-qSOFA AUC (95% CI)** | **ΔAUC** | **DeLong *P*** | **Bootstrap *P*** |
| --- | --- | --- | --- | --- | --- | --- | --- | --- |
| **No vasopressor**  (non-shock) | In-hospital mortality | 2,491 | 69 | 0.79 (0.74–0.84) | **0.82** (0.78–0.87) | 0.03 | 0.0011 | 0.0016 |
|  | ICU admission | 2,491 | 142 | 0.82 (0.80–0.85) | **0.86** (0.83–0.88) | 0.03 | < 0.001 | < 0.001 |
| **Vasopressor**  (shock proxy) | In-hospital mortality | 362 | 127 | 0.54 (0.48–0.59) | 0.54 (0.48–0.60) | 0.01 | 0.6132 | 0.6119 |
|  | ICU admission | 362 | 250 | 0.57 (0.51–0.64) | 0.60 (0.55–0.66) | 0.03 | 0.0535 | 0.0464 |

Abbreviations: AUC, area under the receiver operating characteristic curve; CI, confidence interval; ΔAUC, difference in AUC (SI-qSOFA minus qSOFA).* For the no vasopressor subgroup, ICU admission analysis: DeLong P = 2.63×10⁻¹⁴, Bootstrap P = 3.66×10⁻¹⁴.

Supplementary Figure 1. LASSO regression for in-hospital mortality and ICU admission.

(a, b) Cross-validated binomial deviance (mean ± 1 SE) against log(λ) from 10-fold cross-validation for in-hospital mortality (a) and ICU admission (b); the red and green dashed vertical lines denote λ.min and λ.1se, respectively. (c, d) LASSO coefficient paths of the candidate covariates against log(λ) for in-hospital mortality (c) and ICU admission (d); solid colored lines denote covariates retained at the 1-SE criterion, and grey dashed lines denote covariates shrunk to zero. In all panels, SI-qSOFA was forced into the model without penalization (penalty.factor = 0) and was retained at both λ.min and λ.1se with a consistently positive coefficient (24 and 14 covariates selected for mortality; 11 and 6 for ICU admission, respectively), supporting the robustness of its independent association.
